# The comparative effectiveness of statin therapy in selected chronic diseases compared with the remaining population

**DOI:** 10.1186/1471-2458-12-712

**Published:** 2012-08-30

**Authors:** Xia Sheng, Michael J Murphy, Thomas M MacDonald, Li Wei

**Affiliations:** 1 Medicines Monitoring Unit, Division of Medical Sciences, Ninewells Hospital & Medical School, University of Dundee, Dundee, DD1 9SY, UK; 2 Department of Biochemical Medicine, Division of Clinical & Population Sciences & Education, Ninewells Hospital & Medical School, University of Dundee, Dundee, DD1 9SY, UK

**Keywords:** Statins, Total cholesterol, Cardiovascular, Mortality, Chronic diseases

## Abstract

**Background:**

Total cholesterol (TC) concentration is the most commonly used measure of statin efficacy in the UK. This study aimed to evaluate the effectiveness of statins in lowering TC, cardiovascular events (CV) and mortality five common chronic diseases (chronic obstructive pulmonary disease (COPD), osteoarthritis (OA), rheumatoid arthritis (RA), chronic kidney disease (CKD), and diabetes mellitus (DM)) and to compare effectiveness with the rest of the population not recorded as having these diseases.

**Methods:**

A population-based cohort study was conducted in Tayside population who had at least two TC measurements between 1993 and 2007. There were 12,140 patients with chronic diseases and 9,481 patients in the rest of the population not recorded as having these chronic diseases. The main outcomes were TC change from baseline, CV events and all-cause mortality.

**Results:**

Statin-associated TC reductions varied from 15% to 28% with baseline value of between 5.1 and 5.9 mmol/L in the primary prevention (PP) and from 7% to 23% with baseline value of 4.5 to 5.2 mmol/L in the secondary prevention (SP) among chronic diseases patients. In the rest of the population, TC reductions with statins were 31% in PP and 28% in SP with baselines of 6.3 mmol/L and 5.3 mmol/L, respectively (test of heterogeneity with chronic disease groups: p < 0.001). A notional reduction of 0.5 mmol/L in TC predicted variable reductions in incident CV events of 30% in RA, 19% in CKD, and 20% in DM, and recurrent CV events by 62% in COPD, 16% in CKD, and 19% in DM. The corresponding figures for the rest of population were 12% for incident CV events and 17% for the recurrent CV events, respectively. Risk reductions for all-cause mortality varied from 20% to 36% in PP and from 18% to 40% in SP, except in OA or RA patients in the chronic diseases and 11% in PP and 16% in the rest of population (test of heterogeneity: p > 0.05).

**Conclusions:**

The effectiveness of statins in common chronic diseases varied. With the exception of diabetes, statins tends to be less effective in patients with the chronic diseases compared with the rest of the study population. Changes in TC with statins appear not to correlate well with the changes in cardiovascular events and all-cause mortality.

## Background

Chronic disease now accounts for the majority of global morbidity and mortality [1,2]. The prevalence of chronic obstructive pulmonary disease (COPD) is 9-10% based on spirometry testing [3] and is expected to be the fourth leading cause of death in the world by 2030 [4,5]. At least 2.8% of the population (171 million) in the world suffer from diabetes mellitus (DM) in 2000 and this number is estimated to double by 2030 [6]. Chronic kidney disease (CKD) affects over 500 million people worldwide [7]. The prevalence of CKD is 7.2% in those aged 30 years or older and varies from 23.4% to 35.8% in persons aged 64 years or older [8]. Rheumatoid arthritis (RA) has an estimated prevalence of 0.5–1.0% [9]. Osteoarthritis (OA) is the most common form of arthritis and the leading cause of chronic disability worldwide [10]. The prevalence of OA increases with age and 12% of people over the age of 65 have symptomatic knee OA [11]. In a significant proportion of patients with these chronic diseases, statins would be indicated for the prophylaxis of increased cardiovascular (CV) risk [12-21]. Certainly, dyslipidemia is associated with an increased CV risk in patients with chronic diseases [22-24].

The ability of statins to reduce cardiovascular risk by 21% per mmol of low density lipoprotein cholersterol (LDL-C) reduction was established in trial populations [25]. However, in the UK, clinicians usually make treatment decisions based on total cholesterol (TC), rather than LDL cholesterol (although the latter can in certain circumstances be calculated), sometimes using high density lipoprotein concentration (HDL-C) measurements as well. We have previously shown that TC reduction with lipid-lowering drugs predicts outcomes in the statin trials almost as well as LDL-C and that TC can be used as a reasonable measure of statin efficacy in the absence of LDL-C [26,27]. We set out to compare the effect of statins on total cholesterol-lowering, CV events, and all-cause mortality in patients with five chronic diseases (COPD, OA, RA, CKD, or DM) using the rest of the population who had no record of having these diseases as the referent group.

## Methods

We performed a population-based longitudinal study using the Medicines Monitoring (MEMO) unit record-linkage database in Tayside, Scotland. This study was approved by Tayside Research Ethics Committee and the Tayside Caldicott Guardians. The study population consisted of residents of Tayside who were registered with a general practitioner between January 1993 and December 2007 and remained residents in Tayside or died during the study period. Study subjects were the Tayside population who had at least two different TC measurements separated by at least 30 days of follow-up. They were divided into chronic disease groups and the rest of the population who did not have these chronic diseases. The chronic disease groups included patients with a primary diagnosis of COPD, OA, RA, CKD, or DM between 1993 and 2007. COPD patients were identified from The Tayside Allergy and Respiratory Disease Information system (TARDIS) and Scottish Morbidity Record (SMR01) which are centralised records of all Scottish hospital admission diagnoses; OA or RA patients were identified from the regional Arthritis dataset, SMR01, and patients with disease-modifying anti-rheumatic drugs (DMARDs) use in the prescription database; CKD patients were identified from SMR01 and the regional biochemistry database (serum creatinine of 220 μmol/L or higher); DM patients were identified from Diabetes Audit and Research in Tayside Scotland (DARTS) database which is derived from the Scottish care information diabetes collaboration (SCI-DC) [28]. SMR1 data were available from 1989 onward. The date of their first diagnosis of each disease was used as the entry date in chronic disease group. A frequency-matched calendar year method was used to allocate an entry date for the rest of the population. TC measurements were obtained from the regional biochemistry database. Subjects were categorized into statin-exposed and statin-unexposed groups according to statin use status during the follow-up. Subjects were also classified into primary prevention (PP) and secondary prevention (SP) cohorts according to whether they had established CV disease prior to the entry date.

The main outcomes were TC change calculated as TC at the baseline minus TC at the end of follow up, the incident (new) or recurrent Anti-platelet Trialist’s Collaboration (APTC) events of non-fatal myocardial infarction (MI), non-fatal stroke, or vascular death, the individual components of APTC, and all-cause mortality during the follow-up. All-cause mortality data were obtained from the General Register Office for Scotland. Cox regression models with a time-dependent variable of statins to avoid immoral time bias were employed to assess the risk of APTC events or all-cause mortality and adjusted for potential confounders including age, gender, socioeconomic status, TC concentration change, co-morbidities, and concurrent use of medications. Cox model assumptions were checked prior to analysis. To determine the presence of heterogeneity across different study populations (different chronic diseases and the rest of the population), the heterogeneity test (Cochrane's χ^2^ test (Q-statistics) was performed [29]. ‘Dose equivalents’ of simvastatin was used for other statin treatments in order to calculate the mean daily dose. All analyses were carried out using SAS version 9.1.

## Results

### Statins in chronic diseases

In total, 9,955 patients in the PP cohort (6,037 in the statin-exposed group and 3,918 in the statin-unexposed group) and 2,185 patients in the SP cohort (1,427 in the statin-exposed group and 758 in the statin-unexposed group). The PP cohort consisted of 1,274 COPD, 1,269 OA, 430 RA, 998 CKD, and 5,984 DM patients, in which 617 (48.4%), 696 (54.8%), 181 (42.1%), 442 (44.3%), and 4,101 (68.5%) were in the statin-exposed groups, respectively. The SP cohort included 443 COPD, 247 OA, 78 RA, 704 CKD, and 713 DM patients, in which there were 292 (65.9%), 175 (70.9%), 60 (76.9%), 386 (54.8%), and 514 (72.1%) in the statin-exposed groups, respectively. The mean follow-up years in PP were 3.08 in COPD patients, 3.61 in OA patients, 3.90 in RA patients, 3.61 in CKD patients, and 4.65 in DM patients in the statin-exposed groups, and were 2.51, 2.69, 3.14, 2.51, and 3.50 in the statin-unexposed groups, respectively. Correspondingly, the mean follow-up periods in SP were 3.05, 2.88, 3.15, 2.92, and 4.32 in the statin-exposed group, and were 2.26, 2.72, 2.72, 1.91, and 3.13 in the statin-unexposed groups.

**Figure 1:**
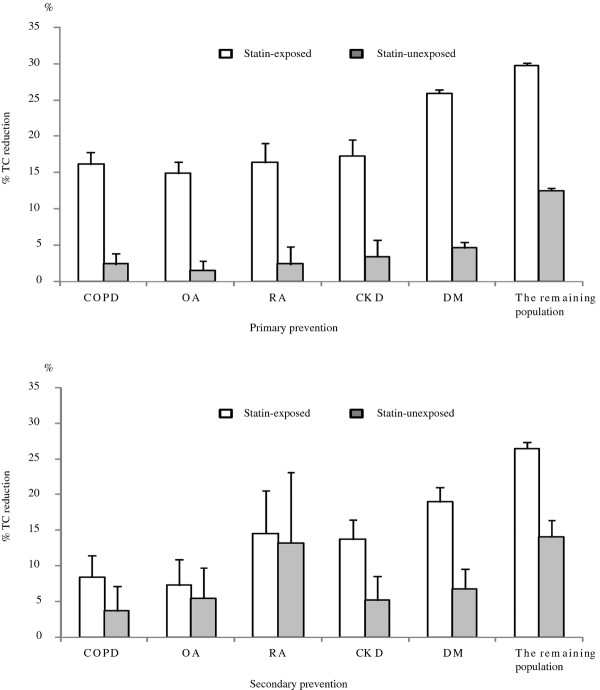
TC concentration changes in primary prevention and secondary prevention patients.

Table1 shows the baseline characteristics of subjects. TC concentrations ranged from 5.11 mmol/L to 5.90 mmol/L in PP and from 4.54 mmol/L to 5.20 mmol/L in SP (P < 0.001 between chronic diseases in both PP and SP). Statin-associated TC reductions varied from 15% to 28% in PP and from 7% to 23% in SP (Figure1). Diabetic patients had the most pronounced TC reduction with statins irrespective of PP or SP. TC changes in the statin-unexposed groups were much lower than those in the statin-exposed groups in both PP and SP (Figure1).

**Table 1:** Baseline characteristics in chronic diseases patients and the rest of the population

	**COPD n (%)**		**OA n (%)**		**RA n (%)**		**CKD n (%)**		**DM n (%)**		**The rest of the population n (%)**	
	***PP***	***SP***	***PP***	***SP***	***PP***	***SP***	***PP***	***SP***	***PP***	***SP***	***PP***	***SP***
**Statin-exposed**	617(48.4)	292(65.9)	696(54.8)	175(70.9)	181(42.1)	60(76.9)	442(44.3)	386(54.8)	4101(68.5)	514(72.1)	4574(57.4)	1315(86.6)
**Age** (years) (mean SD)	68.5(8.8)	70.2(9.0)	68.5(8.9)	70.8(9.6)	63.9(11.5)	68.1(9.9)	66.7(14.1)	73.6(10.5)	60.9(11.8)	67.3(10.5)	64.2(11.1)	67.1(10.9)
**Male**	297(48.1)	169(57.9)	295(42.5)	95(54.6)	46(25.4)	29(48.3)	248(56.4)	239(62.2)	2079(51.1)	323(63.0)	2182(47.7)	824(62.7)
**Baseline TC** (mmol/L) (mean SD)	5.30(1.20)	4.68(1.10)	5.30(1.19)	4.54(1.07)	5.54(1.10)	4.95(1.28)	5.11(1.40)	4.85(1.44)	5.90(1.26)	5.20(1.36)	6.03(1.26)	5.28(1.13)
**Simvastatin daily dose** (mg) (mean SD)	29(19)	29(20)	27(16)	35(27)	28(18)	33(25)	27(18)	26(17)	27(16)	30(24)	28(19)	30(22)
**Social economic status**												
1 (most deprived)	285(46.1)	126(43.2)	141(20.3)	50(28.6)	46(25.4)	18(30.0)	108(25.2)	106(28.5)	1015(25.5)	125(24.9)	837(18.7)	322(25.1)
2-4	254(42.1)	130(45.2	402(58.9)	90(52.6)	100(56.8)	35(58.3)	244(56.9)	210(56.9)	2275(57.1)	310(61.6)	2681(59.8)	760(59.1)
5 (most affluent)	64(10.4)	31(10.6)	139(20.0)	31(17.7)	30(16.6)	7(11.7)	77(18.0)	56(15.1)	693(17.4)	68(13.5)	964(21.5)	203(15.8)
**Concurrent use of drugs**												
Analgesics	453(73.4)	222(76.0)	591(84.9)	156(89.1)	153(84.5)	55(91.7)	303(68.6)	279(72.3)	2293(55.9)	335(65.2)	1897(41.5)	666(50.7)
Positive inotropic drugs	30(4.9)	49(16.8)	17(2.4)	6(3.4)	3(1.7)	7(11.7)	40(9.1)	89(23.1)	174(4.2)	76(14.8)	106(2.3)	62(4.7)
Diuretics	386(62.6)	183(62.7)	416(59.8)	100(57.1)	97(53.6)	39(65.0)	313(70.8)	311(80.6)	1932(47.1)	325(63.2)	2082(45.5)	569(43.3)
Beta-adrenoceptor blocking drugs	97(15.7)	78(26.7)	290(41.7)	94(53.7)	57(31.5)	41(68.3)	204(46.2)	208(53.9)	1456(35.5)	299(58.2)	1816(39.7)	776(59.0)
Hypertension and heart failure	373(60.5)	213(73.0)	431(61.9)	124(70.9)	82(45.3)	48(80.0)	303(68.6)	272(70.5)	2712(66.1)	390(75.9)	2135(46.7)	781(59.4)
Nitrates & calcium-channel blockers	393(63.7)	235(80.5)	415(59.6)	130(74.3)	99(54.7)	51(85.0)	301(68.1)	302(78.2)	1951(47.6)	390(72.9)	2202(48.1)	947(72.0)
Anticoagulants	43(7.0)	49(16.8)	56(8.1)	13(7.4)	13(7.2)	13(21.7)	64(14.5)	104(26.9)	237(5.8)	103(20.0)	227(4.9)	97(7.4)
Antiplatelet drugs	400(64.8)	244(83.6)	423(60.8)	152(86.9)	93(51.4)	45(75.0)	242(54.8)	297(76.9)	2039(49.7)	421(81.9)	2329(50.9)	1107(84.2)
Corticosteroids	457(74.1)	210(71.9)	208(29.9)	46(26.3)	109(60.2)	32(53.3)	163(36.9)	127(32.9)	1093(26.7)	148(28.8)	942(20.6)	260(19.8)
NSAID drugs	180(29.2)	63(21.6)	342(49.1)	71(40.6)	110(60.8)	34(56.7)	91(20.6)	77(20.0)	1586(38.7)	166(32.3)	1292(28.3)	323(24.6)
**Co-morbidity**												
Angina, TIA, heart failure	56(9.1)	142(48.6)	34(4.9)	17(9.7)	6(3.3)	9(15.0)	33(7.5)	140(36.3)	169(4.1)	109(21.2)	187(4.1)	508(38.6)
Diabetes mellitus	111(18.0)	33(11.3)	131(18.8)	14(8.0)	36(19.9)	11(18.3)	67(15.2)	60(15.5)	-	-	-	-

The crude event rates per 1000 person-years (PYs) for each outcome are shown in Additional file 1: Appendix 1. CKD patients taking statins had the highest event rates for most outcomes in both PP and SP. DM patients taking statins had the lowest event rates in PP and OA patients taking statins had the lowest event rates in SP.

**Figure 2:**
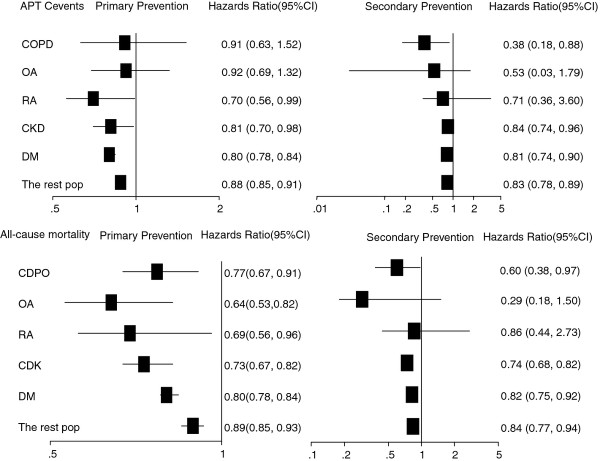
The adjusted hazards ratios of APTC and all-cause mortality in primary and secondary prevention.

**Figure 3:**
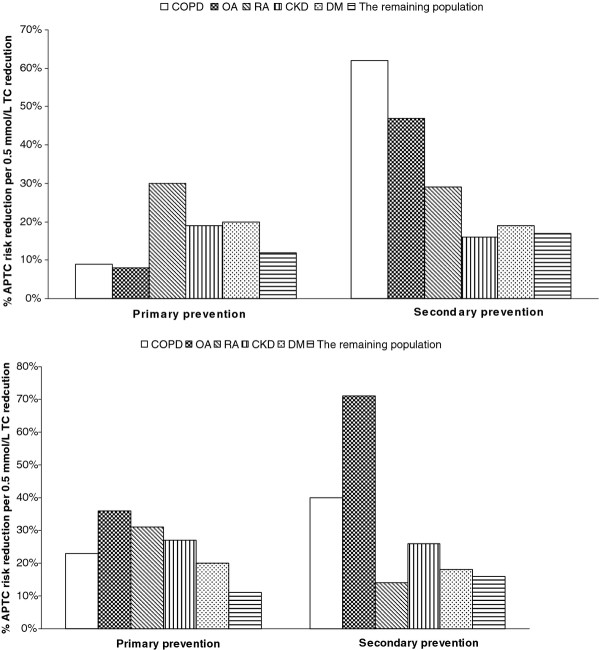
The proportional risk reduction of APTC events and all-cause mortality per 0.5 mmol/L TC concentration reduction.

Figure2 shows the predicted proportional hazard ratio for each outcome per notional 0.5 mmol/L statin-associated TC reduction (in order to avoid negative numbers, hazards ratios are given per 0.5 mmol/L TC reduction). For the incident APTC events, 0.5 mmol/L TC reduction with statins translated into 30% reduction in RA patients, 20% in DM patients, and 19% in CKD patients, but no effect in COPD or OA patients. For recurrent APTC events, a 0.5 mmol/L TC fall resulted in 62% reduction in COPD, 19% in DM, and 16% in CKD, but no effect in OA or RA (Figure3). Risk reduction in incident non-fatal MI or incident non-fatal stroke was observed in DM patients (both 18%), but was not seen in other chronic disease patients. Risk of recurrent non-fatal MI was reduced by 30% in CKD and 24% in DM. Risk of recurrent non-fatal stroke was reduced by 35% in CKD patient. Risk reduction of CV mortality was 37% in OA, 20% in CKD, and 19% in DM in PP, and 65% in COPD, 24% in CKD, and 25% in DM in SP ( Additional file 1: Appendix 2). A 0.5 mmol/L TC reduction was associated with reductions in all-cause mortality of about 23% in COPD, 36% in OA, 31% in RA, 27% in CKD, and 20% in DM in PP, and 40% in COPD, 26% in CKD, and 18% in DM in SP (Figure2).

### Statins in the rest of the population

7,964 subjects (4,574 statin-exposed and 3,390 statin-unexposed) were included in PP with a mean follow up of 3.07 years in the statin-exposed group and 2.65 in the statin-unexposed group and 1,517 subjects (1,315 statin-exposed and 202 statin-unexposed) in SP with a mean follow up of 2.89 and 2.37 years, respectively. Statin-associated TC concentration fell by 31% from baseline of 6.03 mmol/L in PP and 28% from 5.28 mmol/L in SP (Figure1). Approximately 12% of TC reduction was also observed in the statin-unexposed group.

The crude event rates per 1000 PYs were 14.8 (95%CI 12.9-16.9) for incident APTC events and 43.4 (95%CI 37.3-50.6) for recurrent APTC events in the statin-exposed group. And the crude mortality rate were 12.2 (95%CI 10.5-14.2) in PP and 33.8 (95%CI 28.4-40.3) in SP ( Additional file 1: Appendix 1). Statin treatment reduced APTC events, APTC components, or all-cause mortality by approximately 12% in PP and by approximately 17% in SP per 0.5 mmol/L TC reduction (Figure2 and Additional file 1: Appendix 2).

### Comparison between chronic disease patients and the rest of the population

Baseline TC concentrations in chronic disease patients were lower than those in the rest of the population in the statin-exposed groups (both PP and SP: P < 0.001). Compared with chronic diseases patients, the mmol/L fall of TC concentration and the percentage fall of TC with statin use were larger in the remaining population (both PP and SP: P < 0.001). The heterogeneity of the proportional risk reduction in incident APTC events (p < 0.01) and all-cause mortality in PP (p < 0.0001) per 0.5 mmol/L TC reduction was present across chronic disease patients and the rest of the population, but not in recurrent APTC events (p = 0.53), incident non-fatal MI (p = 0.66), recurrent non-fatal MI (p = 0.39), incident non-fatal stroke (p = 0.60), recurrent non-fatal stroke (p = 0.10), CV mortality in PP (p = 0.16), CV mortality in SP (p = 0.22), and all-cause mortality in SP (p = 0.12).

## Discussion

We have examined the effectiveness of statins in chronic disease patients compared with the remainder of the population. With the exception of DM, statin-associated TC reductions in chronic disease patients were in general smaller than those in the rest of the population. Use of statins was associated with improved survival in these chronic disease patients for the primary prevention of CV events and in COPD, CKD, or DM patients for the secondary prevention of CV events. The risk reductions in APTC events and all-cause mortality in PP were significantly different across chronic disease patients and the rest of the population, but were not heterogeneous in SP.

The majority of previous studies focused on LDL-C concentration when evaluating the effect of statins in disease population. In this study, total cholesterol rather than LDL-C concentration was used to investigate the effect of statins on cholesterol changes in different chronic disease populations. There were several reasons: firstly, in the UK clinicians usually make statin titration decisions based on TC plus or minus HDL-C measurements. Secondly, LDL-C can be measured from the blood but it is expensive. So LDL-C is rarely measured in clinical practice and is usually calculated instead. A mathematical equation called Friedewald equation (LDL = TC-HDL-TG/2.17 (mmol/L) is used to calculate LDL-C using values for total cholesterol, HDL-C and TG. When calculating LDL-C with the equation, it requires a fasting TG measurement [30]. Thirdly, in Tayside population there were approximately 15% of patients (COPD, OA, RA, or CKD) with at least two separate TC measurements and 35% in diabetic patients. HDL-C concentration had similar measurements as TC concentration in MEMO database. However, there were very few TG measurements in these populations. This resulted in a large number of missing records for calculating LDL-C concentration and the effect of statins on LDL-C could not be investigated in this study.

In chronic disease patients exposed to statins, baseline TC concentrations were lower than those in the rest of the population. There are several possible explanations for this. Firstly, the average age in the chronic disease groups was higher than those in the rest of the population in SP (p < 0.001), and cholesterol concentrations decrease with age in older men and women [31,32]. Secondly, the rest of the population group was defined as population with at least two different TC measurements. These people were more likely to have other medical conditions such as hyperlipidmia, MI, and possibly to have higher baseline TC than the general population. So they were not completely representative of the general population. Thirdly, decreased TC concentration is seen with chronic disease or inflammation in the elderly [33-40]. Fourthly, low TC concentration is found with malnutrition or poor health status in elderly persons [33,35]. This appears to be related to lower baseline TC in SP than in PP. Fifthly, it could be also due to greater tendency of physicians to begin statins therapy earlier in patients with several risk factors of CV events. Finally, different mechanisms in chronic diseases may lead to different baseline TC and different TC reductions after statin therapy.

In addition, the impact of statins in chronic disease patients was less than that in the general population. One explanation was likely that the baseline TC concentrations did not reflect usual levels in these disease populations. The timing of lipid estimations can affect the results sometimes quite markedly. Cholesterol tests include fasting and non-fasting (random) blood samples. A recent study compared fasting and non-fasting TC and HDL-C concentration in adults and found that there were statistically significant differences between fasting and nonfasting results for total cholesterol, but no significant difference between non-fasting HDL-C and fasting HDL-C [41]. TC concentrations were slightly higher in the non-fasting state, but fasting and non-fasting values were highly correlated [41]. Therefore it is likely that many of lipid collections were non-fasting and performed during inter current illness, which result in different responses to statins in these chronic disease patients. Although there was a difference in TC, it was not clinically significant in diabetic or non-diabetic patients [42].

Average daily doses of statins in chronic disease groups were similar to those in the rest of the population in both PP and in SP. This suggested that differential TC reductions with statin use across the disease groups were not due to different statin doses. In secondary prevention there was no heterogeneity in the risk reduction across different study populations. This might be because the data were sparse in some chronic diseases with resulting low statistical power. In addition, the heterogeneous effects of standardized dose of simvastatin may be affected by the type of lipid abnormality and heterogeneity in differing disease states. For example, diabetic dyslipidemia consists of low HDL-C concentrations, increased TG concentrations, and postprandial lipemia, which is not captured by a focus on TC concentration [43]. In CKD patients, depressed HDL-C and increased TG are the major lipid abnormalities and LDL-C showed a ‘J-curve’ with respect to severity of disease [44]. Some evidence suggests that LDL-C is not increased and LDL-C is not strongly correlated with outcome in CKD [45].

Our study found that although there was less TC reduction with statins in some chronic disease groups than those in the rest of population, more benefit on the risk reduction of the outcomes was observed in some chronic disease groups, perhaps reflecting differential impacts of the pleiotropic anti-inflammatory effect of statins in chronic diseases. For example, statins have been suggested to have pleiotropic (anti-inflammatory) effects in patients with inflammatory diseases such as COPD and rheumatoid arthritis [46-48].

The beneficial effects of statins have been seen in clinical trials and observational studies in COPD, RA, CKD and diabetes [49-57]. Mancini et al reported that statin use exhibited a reduced MI risk ratio (RR 0.48 95% CI 0.39-0.59) in COPD patients with coronary revascularization (high CV risk cohort) [49]. Daily use of 20 mg or 40 mg simvastatin was associated with the range from 18% to 24% TC reduction and exhibited an improvement in vascular function in RA patients [51-53]. A meta-analysis that examined fifty RCTs and found that TC concentration was significantly lower by 19% with statins than with placebo in CKD patients with established CVD [56]. A 21% risk reductions with lipid-lowering drugs in both incident and recurrent major coronary events in diabetic patients has been reported in another meta-analysis [57]. TC concentration showed a decrease of 15-20% in diabetic groups, which was a little smaller than that in our findings. In general, although there were some differences in the complexity of study design and patients selection between these studies and ours, they were in general agreements with our findings in patients with COPD or CKD or diabetes mellitus.

This is the first population-based study to investigate the comparative effectiveness of statins across chronic diseases patients and the rest of the population.While some randomized clinical trials provided the evidence of statin efficacy in an ideal setting in these chronic disease patients, our study assessed the effectiveness of statins under usual care setting. The study by using healthcare utilization databases has good external validity. In addition, we took into account an extensive list of covariates in adjusting for potential confounders. However, there are some limitations in our study. Our study may be influenced by potential unmeasured or immeasurable confounders such as cofounders related to the disease inherent progress or some other confounders of smoking status and alcohol consumption. We included the effect of statins on cholesterol lowering in five chronic diseases, but we did not study other chronic diseases such as chronic hepatitis, autoimmune diseases, etc. Another limitation of our study is the relatively small number of OA or RA patients with prior CV disease and resulting limited statistical power to provide meaningful results. We assumed if a patient dispensed his/her statin prescription he/she would be adherent to the treatment. However, we have no way of knowing true adherence. Furthermore comparative effectiveness studies in larger populations are required to confirm these relationships (e.g. the Trial of Atorvastatin for the primary prevention of cardiovascular events in patients with rheumatoid arthritis (TRACE-RA)). Also the effects of statins were not separately studied for men and women in our study. Gender differences might help to explain differential effects of statins in chronic diseases. Adherence and persistence of statins might also affect the effectiveness of statins and could vary between chronic diseases. Further studies should also study individual statins, smoking status, alcohol consumption, and other possible confounders in different populations.

## Conclusion

The cholesterol lowering effect of statins among chronic diseases patients was generally less than that in the rest of the study population and showed considerable heterogeneity. The proportional risk reduction in cardiovascular events and all-cause mortality with statins per 0.5 mmol/L TC reduction also varied across different chronic disease groups compared with the rest of the population in PP, but there was no heterogeneity in SP. There was also an apparent disconnection between reductions in TC and outcome benefits that warrants further study. For practising clinicians, there appears to be meaningful heterogeneity in the effectiveness of statins between different disease groups which warrants further study.

## Competing interests

All authors declare: no support from any organisation for the submitted work; no financial relationships with any organisations that might have an interest in the submitted work in the previous 3 years; no other relationships or activities that could appear to have influenced the submitted work.

## Authors’ contribution

XS carried out the statistical analysis under the supervision of LW and wrote the first draft of the paper. LW, MJM, TMM were involved in the design of the study, interpretation of results and re-drafting of the paper. LW is guarantor for this study. All authors read and approved the final manuscript.

## Pre-publication history

The pre-publication history for this paper can be accessed here:

http://www.biomedcentral.com/1471-2458/12/712/prepub

## Supplementary Material

Additional file 1 **Appendix 1.**The crude event rates for the outcomes in chronic diseases patients and the rest of the population. Appendix 2. The adjusted hazards ratios of outcomes with statin use in PP and SP. Click here for file

## Author details

^1^Medicines Monitoring Unit, Division of Medical Sciences, Ninewells Hospital & Medical School, University of Dundee, Dundee DD1 9SY, UK. ^2^Department of Biochemical Medicine, Division of Clinical & Population Sciences & Education, Ninewells Hospital & Medical School, University of Dundee, Dundee DD1 9SY, UK.
