# Supplementary material for: The comparative effectiveness of statin therapy in selected chronic diseases compared with the remaining population
Source: BMC Public Health. 2012 Aug 30;12:712. doi: 10.1186/1471-2458-12-712 (PMC3490740; doi:10.1186/1471-2458-12-712)
Supplement: Additional file 1 — Appendix 1.The crude event rates for the outcomes in chronic diseases patients and the rest of the population. Appendix 2. The adjusted hazards ratios of outcomes with statin use in PP and SP. [file 1471-2458-12-712-S1.doc]

Appendix 1. The crude event rates for the outcomes in chronic diseases patients and the rest of the population

*Data expressed as per 1000 person-years with 95% confidence interval

Appendix 2. The adjusted hazards ratios of outcomes with statin use in PP and SP

|  | **No. of patients/ No. of APTC events** | | **Adjusted HR (95%CI)*** | | | | | | | | | |
| --- | --- | --- | --- | --- | --- | --- | --- | --- | --- | --- | --- | --- |
|  | **statin-exposed**  **group** | **statin-unexposed**  **group** | **APTC events** | **Non-fatal MI** | | **Non-fatal stroke** | | | **CV mortality** | | **All-cause**  **mortality** | |
| *Primary prevention* | | | | | | | | | | | | |
| **COPD** | 617/55 | 657/63 | 0.91(0.63-1.52) | 0.88(0.51-2.90) | | | 0.92(0.64-1.53) | | 0.94(0.62-1.78) | | **0.77(0.67-0.91)** | |
| **OA** | 696/34 | 573/34 | 0.92(0.69-1.32) | 0.94(0.59-1.84) | | | 0.86(0.51-2.10) | | **0.63(0.49-0.92)** | | **0.64(0.53-0.82)** | |
| **RA** | 181/10 | 249/19 | **0.70(0.56-0.99)** | 0.65(0.51-1.15) | | | 0.64(0.48-1.51) | | 0.82(0.55-1.77) | | **0.69(0.56-0.96)** | |
| **CKD** | 442/61 | 556/100 | **0.81(0.70-0.98)** | 0.92(0.66-1.45) | | | 0.87(0.63-1.38) | | **0.80(0.67-0.99)** | | **0.73(0.67-0.82)** | |
| **DM** | 4101/188 | 1883/194 | **0.80(0.78-0.84)** | **0.82(0.78-0.88)** | | | **0.82(0.78-0.89)** | | **0.81(0.77-0.85)** | | **0.80(0.78-0.84)** | |
| **The rest of the population** | 4574/208 | 3390/219 | **0.88(0.85-0.91)** | **0.86(0.82-0.91)** | | | **0.88(0.84-0.94)** | | **0.90(0.84-0.99)** | | **0.89(0.85-0.93)** | |
| *Secondary prevention* | | | | | | | | | | | | |
| **COPD** | 292/42 | 151/42 | **0.38(0.18-0.88)** | | 0.36(0.10-1.89) | | | 0.26(0.07-1.51) | | **0.35(0.16-0.78)** | | **0.60(0.38-0.97)** |
| **OA** | 175/19 | 72/17 | 0.53(0.03-1.79) | | 0.50(0.24-3.49) | | | 0.16(0.07-3.41) | | 0.37(0.24-2.60) | | 0.29(0.18-1.50) |
| **RA** | 60/9 | 18/4 | 0.71(0.36-3.60) | | 0.46(0.34-1.28) | | | - | | 0.61(0.38-1.54) | | 0.86(0.44-2.73) |
| **CKD** | 386/128 | 318/140 | **0.84(0.74-0.96)** | | **0.70(0.57-0.95)** | | | **0.65(0.52-0.98)** | | **0.76(0.67-0.87)** | | **0.74(0.68-0.82)** |
| **DM** | 514/99 | 199/77 | **0.81(0.74-0.90)** | | **0.76(0.68-0.92)** | | | 0.92(0.74-1.29) | | **0.75(0.68-0.87)** | | **0.82(0.75-0.92)** |
| **The rest of the population** | 1315/165 | 202/64 | **0.83(0.78-0.89)** | | **0.81(0.75-0.92)** | | | **0.85(0.77-0.99)** | | **0.83(0.75-0.99)** | | **0.84(0.77-0.94)** |

***** Cox regression models with a time-dependent variable of statins were employed to adjust for potential confounders including age, gender, socioeconomic status, TC concentration change, co-morbidity of angina, TIA, heart failure, or diabetes and concurrent use of medications of analgesics, positive inotropic drugs, diuretics, beta-adrenoceptor blocking drugs, drugs for treating hypertension and heart failure, nitrates & calcium-channel blockers, anticoagulants, antiplatelet drugs, corticosteroids, and NSAID drugs.
